# Clinical analysis of the tooth-implant papilla for two narrow-diameter titanium-zirconium implants in the anterior area: prospective controlled clinical study

**DOI:** 10.1186/s12903-024-04075-2

**Published:** 2024-03-05

**Authors:** Paola Herrera-Pérez, Ana María García-De-La-Fuente, Eztizen Andia-Larrea, Xabier Marichalar-Mendia, José Manuel Aguirre-Urizar, Luis Antonio Aguirre-Zorzano

**Affiliations:** 1 University of the Basque Country (UPV/EHU), Biscay, Spain; 2 Research Group: GIU21/042Department of StomatologyFaculty of Medicine and Nursing, University of the Basque Country (UPV/EHU), Barrio Sarriena S/N, 48940 Leioa, Biscay Spain; 3 International University of Catalunya, Barcelona, Spain; 4 Department of Stomatology, University of the Basque Country (UPV/EHU), Biscay, Spain; 5 Research Group: GIU21/042, Department of Nursery I, University of the Basque Country (UPV/EHU), Biscay, Spain

**Keywords:** Dental implants, Ti-Zr alloy, Single-tooth, Aesthetic area, Incisor, Patient-reported outcomes measures, Prospective study, Prosthodontics

## Abstract

**Background:**

Rehabilitation of the anterior area when the mesio-distal space is reduced is a challenge for the clinician, due to the patient’s anatomical limitations and aesthetic requirements. Narrow Diameter Implants (NDI) are an option of treatment when the standard diameter implant is not possible, but the evidence is scarce. This prospective clinical study aims to analyze the formation of the tooth-implant papilla between the implant and the adjacent natural tooth in the maxillary lateral incisors and mandibular incisors.

**Methods:**

A total of 40 patients treated with NDI, of titanium-zirconium (Ti-Zr) alloy i.e., 2.9 mm Test Group (TG) and 3.3 mm Control Group (CG), were included. The mesiodistal distance between the adjacent natural teeth was used for implant selection, maintaining 1.5 mm between the fixation and the adjacent tooth. Clinical assessment was performed by a clinical examiner at 6 and 12 months after the final prosthesis. The primary variable was the Jemt Papillary Index. Also, implant survival rate (SR), complications, Implant Stability Quotient (ISQ), and patient-reported outcomes measures (PROMs) such as aesthetics, chewing, phonation, comfort, and self-esteem were analyzed.

**Results:**

A significant amount of papilla filling was observed concerning the baseline, with a trend towards more formation of the papilla in the TG, with a JPI score of 3. No significant differences were observed between the two groups regarding implant SR, clinical parameters, and complications. In terms of PROMs, a higher satisfaction in the TG was observed, with significant intergroup differences for aesthetics, comfort, self-esteem, and primary stability ISQ (TG: 59.05 (SD: 5.4) *vs.* CG: 51.55 (SD: 5.7)).

**Conclusions:**

The 2.9 mm diameter Ti-Zr implants achieved a formation of papilla similar to 3.3 mm implants in the anterior region at 12 months of follow-up after the final prosthetic restoration. The use of Ti-Zr implants with a diameter of 2.9 mm to rehabilitate single teeth in areas of the anterior region, where the mesiodistal distance is limited, showed favorable clinical results and a high degree of satisfaction during 1 year of observation similar to 3.3 mm dental implants.

**Trial registration:**

This study was retrospectively registered in ClinicalTrials.gov with the number NCT05642520, dated 18/11/2022.

**Supplementary Information:**

The online version contains supplementary material available at 10.1186/s12903-024-04075-2.

## Introduction

Total or partial edentulism include alterations of oral function and disruptions in the social life and daily activities of patients [1]. Dental implants have become fundamental tools in the replacement of missing teeth [2, 3] and provide aesthetics and functionality. Demand for dental implants has increased in all age groups, including patients > 55 years of age [2, 4].The loss of teeth in the anterior area is related to a decrease in self-esteem and psychosocial [5] well-being. Individuals with such tooth loss avoid participation in social activities because they feel embarrassed to speak, smile, or eat in front of other people, potentially leading to social isolation [6]. In the anterior area, implant rehabilitation is more complex due to anatomical limitations (reduced mesiodistal space, root proximity, narrow ridges, proximity of vascular and neurological structures in mandibular region) [3, 7–12]. One of the intrasurgical complications in the mandibular interforaminal region is the possibility of severe hemorrhage due to the perforation of the lingual cortex where mandibular lingual foraminas and mucosal vessels could be located below the inferior incisors [12]. Also, the aesthetic requirements of patients [13] should be a key factor. Other factors could influence in the success of this treatment to obtain a good aesthetic result in terms of soft tissue and prosthetic rehabilitation, such as the optimal placement of the implant [13], the distance of the bone alveolar ridge to the interproximal contact point [14], the form of the final restoration [15].

Therefore, the rehabilitation of the mandibular incisors and/or maxillary lateral incisors (MI/MxLI) is a challenge for clinicians. Immediate dental implants could be a good treatment choice, but this kind of surgery is not always possible [16, 17].

Hence, narrow-diameter implants (NDIs) have been developed as an alternative treatment for these complex scenarios [3, 18]. These implants minimize the risk of damaging vital structures and decrease the need to perform regenerative surgeries associated with conventional-dimension implants [19], which are associated with longer treatment times and costs for patients as well as an increase in postoperative morbidity [20, 21].

The evidence regarding NDIs is limited, especially for implants smaller than 3 mm [18, 22]. These implants showed higher fracture rates than Titanium implants [23], so a titanium-zirconium (TI-Zr) alloy was developed to minimize this risk. These implants showed greater biomechanical strength (> 15%) than Grade IV Titanium implants [24, 25]. For NDIs > 3 mm, the survival rates (SRs) [3, 18] are similar to those for standard diameter implants [3, 26, 27]. However, for implants < 3 mm, SRs are lower [18, 28] although supporting evidence for these observations is limited [18]. Notably, most studies of NDIs < 3 mm have been carried out with mini-implants (< 2.5 mm), and in edentulous patients treated with overdentures; the SR was attributed to lower resistance to implant and attachment fracture [23, 29] and a smaller contact surface with the bone [30, 31].

Currently, SR is not the most important outcome in the anterior maxillary region. The success of implant treatment is determined by the SR and the peri-implant soft tissues, which must be congruent with the gingiva of the adjacent teeth [32]. In addition, the patient-reported outcome measures (PROMs) [33] with the aesthetic and functional results [34] are important and contribute to outcomes being considered successful [32].

The main objective of this study was to analyze the formation of tooth-implant papilla measured by the Jemt Papillary Index (JPI) [35], in the treatment of MI/MxLI using a 2.9 mm NDI or 3.3 mm NDI. The null hypothesis (H0) was that there were no significant differences in the formation of the tooth-implant papilla between the two types of implants at 12 months of follow-up after the placement of the final prosthetic restoration.

## Materials and methods

### Study design and population and inclusion and exclusion criteria

This was a prospective controlled clinical study (double-blind) with two parallel groups (test group (TG; 2.9-mm NDI) and a control group (CG; 3.3-mm NDI)) with a follow-up of 12 months after the final prosthetic restoration.

The study was performed conducted following STROBE guidelines. The protocol was approved by the Euskadi Drug Research Ethics Committee (CEIm-E) with the code PS2017095 in December 2017. Patients received information about the implant treatment, the advantages and disadvantages of participating in this study. Informed consent was obtained from all participants before the start of the study.

The study included patients from the Master in Periodontology and Osseointegration of the University of the Basque Country (UPV/EHU) and a private clinic, both located in Bizkaia.

The inclusion criteria were as follows:

- patients ≥ 18 years of age;
- absence of single upper lateral incisors o lower incisors with natural adjacent teeth;
- periodontally healthy patients [36]
- patients with history of treated periodontitis
- plaque index [37] ≤ 25%;
- bleeding index [38] ≤ 25%
- non-smokers or light smokers (≤ 10 cigarettes a day)

The exclusion criteria were as follows:

- patients with any systemic condition or disease that may contraindicate the intervention [39]
- patients allergic to titanium and other metals;
- history of radiation therapy to the head or neck;
- uncontrolled diabetes
- pregnant or breastfeeding patients.

### Study group allocation

The implants included in this study were Straumann implants® made of a titanium and zirconium alloy (Roxolid®) with a SLAactive surface® (Institut Straumann AG, Basel, Switzerland). Patient allocation was determined using the mesiodistal width between the two adjacent natural teeth, maintaining at least 1.5 mm between the implant and the adjacent tooth [13]. When this width was between 5.9 mm and 6.3 mm, the patient was included in the 2.9-mm implant group (TG), if this distance was between 6.4 mm and 7.1 mm, the patient was included in the 3.3-mm implant group (CG).

### Control of study bias

The clinical examiner (AMGF) and the biostatistician (XMM) were blinded to the type of implant used. The reproducibility of the clinical examiner (AMGF) was determined by evaluating the presence of papilla (JPI) [35] between a single dental implant and an adjacent natural tooth in 4 patients, at least twice, with a separation of at least 24 h. These implants were not included in the study. An intraclass correlation coefficient > 0.75 was considered acceptable.

### Surgical procedure

Before surgery, periodontal treatment was delivered to all patients who required it. A preoperative radiographic assessment was performed, consisting of a cone beam computed tomography (CBCT), orthopantomography, and periapical radiographs of the edentulous area.

**Fig. 1:**
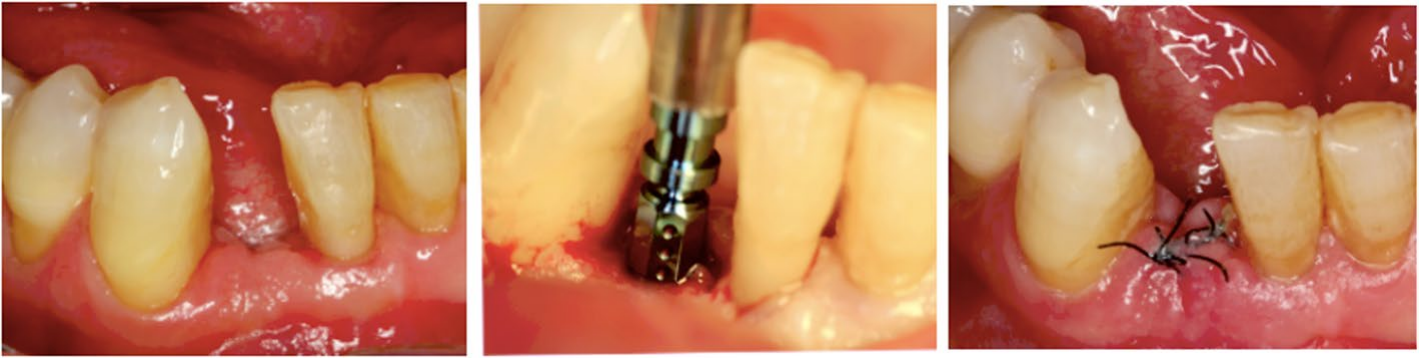
Surgery for implant placement

Before starting surgery, the mouth was rinsed with 0.12% chlorhexidine digluconate and 0.05% cetylpyridinium chloride (Perio-Aid®, Dentaid SL, Barcelona, Spain) for 1 min. The surgical procedure was performed by a single experienced surgeon (PSHP) with more than 10 years of experience. The approach consisted of a full-thickness flap and drilling following the manufacturer's instructions for implant insertion (Straumann®, Institut Straumann AG, Basel, Switzerland) (Fig. 1). The Implant Stability Quotient (ISQ) (Osstell®, Göterborg, Switzerland) was determined using Smartpeg®. This device is screwed directly into the implant and records values ranging from 1 to 10. For each implant, two measurements were taken, and the mean of the two values was recorded as the ISQ. Next, the closure cap for each type of implant (Small CrossFit® or Narrow CrossFit®, Straumann AG, Basel, Switzerland) was placed and sutured with nonabsorbable suture (Supramid®, Laboratorio Aragón, Barcelona, Spain), which was removed after 10 days.

The immediate postsurgical protocol consisted of the administration of the following:

- Amoxicillin (500 mg; Laboratorios Normon SA, Madrid, Spain) every 8 h for 7 days, starting 1 day before the intervention. Clindamycin was administered in patients who were allergic to penicillin (300 mg; Dalacin Pfizer® SL, Madrid, Spain).
- Ibuprofen (400 mg; Kern Pharma, SL, Barcelona, Spain), administered orally every 8 h for 4–5 days.
- Chlorhexidine digluconate (0.12%) and cetylpyridinium chloride (0.05%) mouthwash (Perio-Aid®, Dentaid SL, Barcelona, Spain), 2 times a day for 2 weeks.

### Prosthetic procedure

**Fig. 2:**
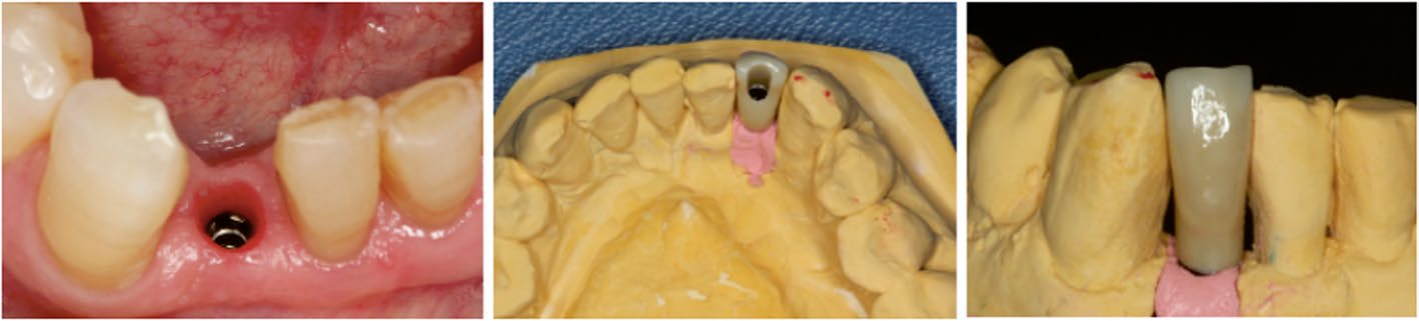
Manufacture of the prosthesis

Before conducting the prosthetic treatment, the ISQ was recorded (Osstell®, Göterborg, Switzerland) in the same way as on the day of surgery. All prosthetic restorations were performed by an experienced implant prosthodontist (EAL), in the same laboratory, 3 months after the surgery. The placement of the final prosthesis was considered the baseline of the study (T0) (Fig. 2).

### Outcome measures

A single experienced, blinded, and previously calibrated examiner (AMGF) recorded the following clinical parameters using a manual periodontal probe (PCP-11, Hu-Friedy®; Chicago, IL, United States). Probing to the closest millimeter was gently performed in each implant and adjacent teeth. The variables were recorded in three different timepoints after the placement of the prosthesis: at baseline(T0), at 6 (T1) and 12 months (T2).

#### Primary outcome

Jemt papillary index (JPI) [35]: the presence of mesial and distal papilla located in the interproximal space between the adjacent natural tooth and a dental implant was recorded: 0 = absence of tooth-implant papilla; 1 = presence of less than 50% of the height of the proximal area occupied by the formation of soft tissue; 2 = there is soft tissue (papilla) in more than 50% of the interproximal space; 3 = complete formation of the papilla in the interproximal space with good aesthetic congruence; and 4 = hyperplastic tooth-implant papilla with irregular soft tissue.

#### Secondary outcomes

- Implant SR: the presence of the implant in the mouth at the time of the assessment.
- Peri-implant Probing Depth (PPD): distance from the gingival margin to the deepest point of the peri-implant sulcus.
- Bleeding on probing (BOP): presence of bleeding after a gentle peri-implant examination.
- Suppuration: the presence of suppuration after a soft peri-implant assessment.
- Modified plaque index (MPI) [40].
- Surgical and prosthetic complications: presence of intense postoperative pain, infection or inflammation, fracture of ceramic, screw loosening, implant or abutment fracture.
- PROMs: patient satisfaction was evaluated using a Likert-type questionnaire in which 5 parameters were evaluated: aesthetics, chewing, phonation, comfort, and self-esteem. The degree of satisfaction for each parameter was recorded using a visual analog scale (VAS) (0–10), where 0 was considered "not at all satisfied" and 10 was "totally satisfied" with the treatment received.

### Sample size calculation

Based on a previous study by Patil et al*.* [41] to detect an effect size of 1.8 and a standard deviation of 1.96, 19 patients per group were needed with an alpha risk of 5% and a statistical power of 80%. This sample size was increased to 40 patients to compensate for possible dropouts. The sample size calculation was performed using the statistical program IBM SPSS v28.

### Statistical analysis

Descriptive univariate analysis was performed. For the qualitative variables, frequency and percentages were used (JPI [35] and sex), for the quantitative variables, mean, standard deviation, and range (probing depth, bleeding on probing, plaque score, suppuration on probing, age, chewing, comfort, esthetic, phonation, self-esteem). The normality of the quantitative variables was evaluated using the Shapiro–Wilk test. For the bivariate analysis, the type of variable was considered: if the two variables were categorical, the chi-square test was used; for dependent variables, the McNemar test was used. The McNemar test is used when we want to compare two dependent and qualitative variables, for example, the JPI index [35] within a group between T0 and T12. For all other variables, the Mann–Whitney U test was used (e.g., age or plaque index between the groups). For the analysis of the JPI in patients with a history of periodontitis or not, the Chi-square test was used.

A *p* value < 0.05 was considered statistically significant. All analyses were carried out using the statistical program IBM SPSS v28, and the graphs were generated in Microsoft Excel.

## Results

### Study sample characteristics

A total of 40 patients were recruited between February 2018 and February 2021 for MI/MxLI replacement. The group consisted of 40 patients (*n* = 40 implants with a length between 8 and 10 mm in all cases), of whom 20 were women (50%); this percentage was the same in both study groups. There were no dropouts during the 12 months of the study, and all patients attended the follow-up visits, allowing verification of an implant SR of 100%.

Most of the patients had a history of periodontitis (TG: 95% vs CG:65%). The mean age of the patients was higher in the TG (67.3 years) than in the CG (59.3 years) (*p* = 0.013). There were smokers in both groups: in the TG, one patient smoked seven cigarettes a day (5%) and in the CG, four patients were smokers (20%), with 3 patients (15%) in this group smoking 10 cigarettes a day.

The most frequent location was the maxillary lateral incisor (*n* = 27), accounting for 67.5% of the sample. Within the TG, the maxillary lateral incisor (*n* = 7, 35%) and the mandibular central incisor (*n* = 7, 35%) were the most frequent locations, followed by the mandibular lateral incisor (*n* = 6, 30%). One hundred percent (*n* = 20) of the locations in the CG involved maxillary lateral incisors.

The initial stability of the implants at the time of surgery was higher in the TG (ISQ = 59.1 ± 5.4, (48–65)) than in the CG (ISQ = 51.6 ± 5.7, (44–65)) (*p* < 0.001). At the time of implant-supported crown placement (T0), stability increased in both groups (TG: ISQ = 75.6 ± 5.2, (63–82) *vs.* CG: ISQ = 73 ± 4.2, (66–83)) without significant differences between the groups. When analyzing the intra-group change for both the TG and CG from the time of implant placement to crown placement, the values increased significantly (*p* < 0.001).

The majority of the patients (65%) showed absence of the incisor more than 1 year ago, and they refused to wear a provisional prosthesis during the healing time after the implant placement. The rest of the patients were provisionally rehabilitated.

**Fig. 3:**
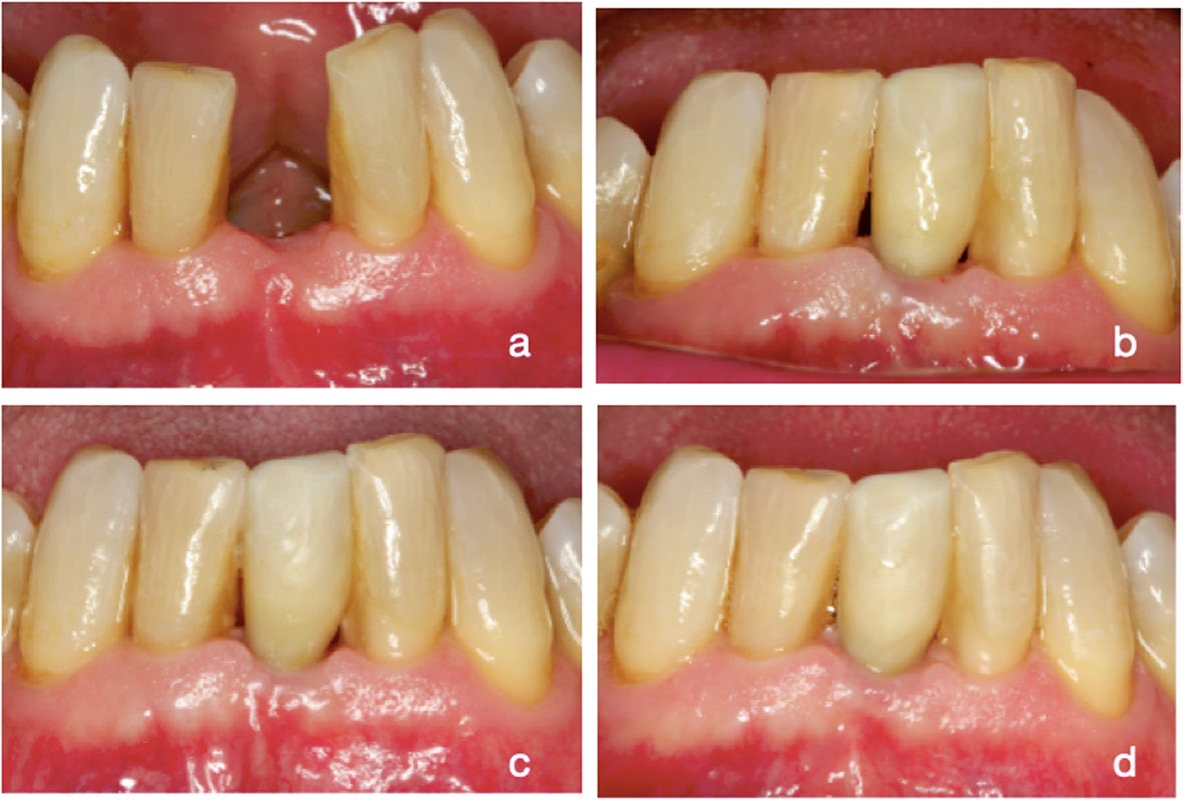
Test group: **a** baseline; **b** placement of the prosthesis; **c** 6 months; **d** 12 months

**Fig. 4:**
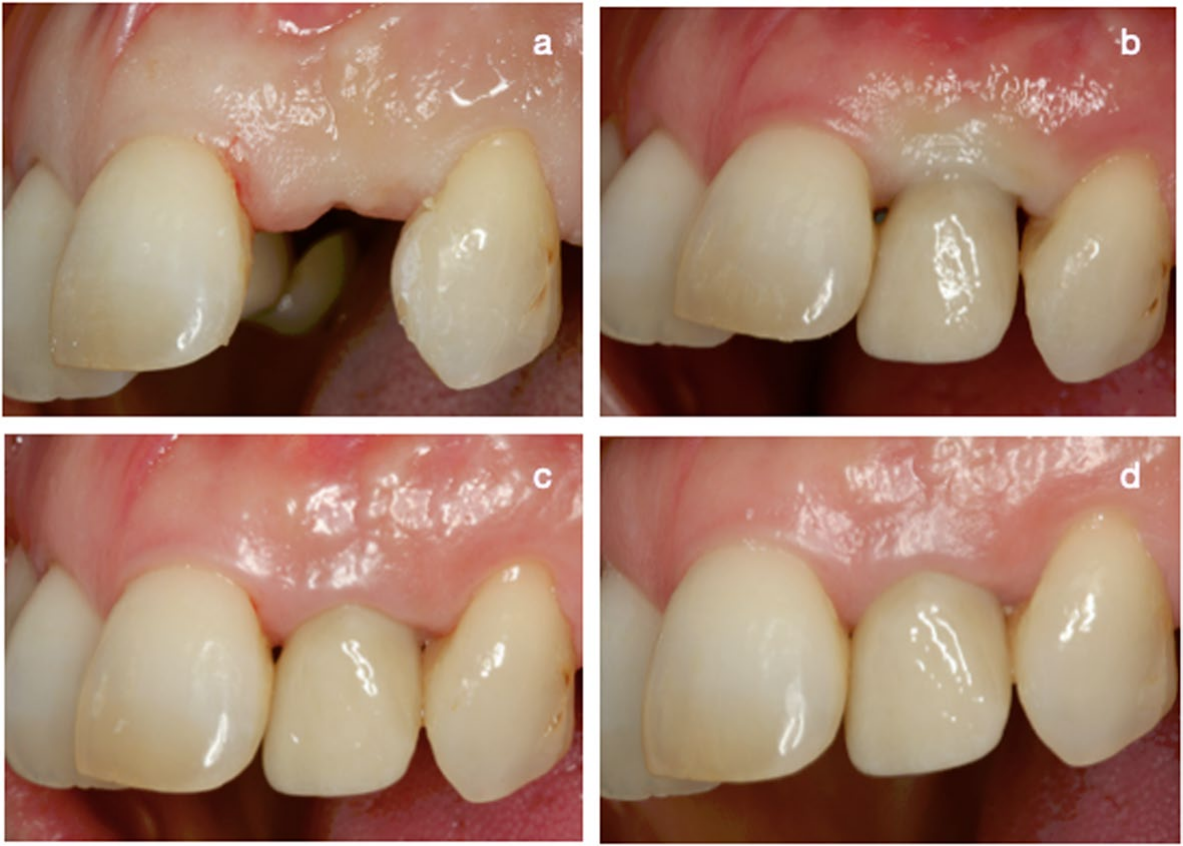
Control group: **a** baseline; **b** placement of the prosthesis; **c** 6 months; **d** 12 months

The clinical characteristics of two patients of TG and CG at baseline, 6 and 12 months are shown in Figs. 3 and 4.

### Analysis of the Jemt Papilla Index (JPI) [35]

**Fig. 5:**
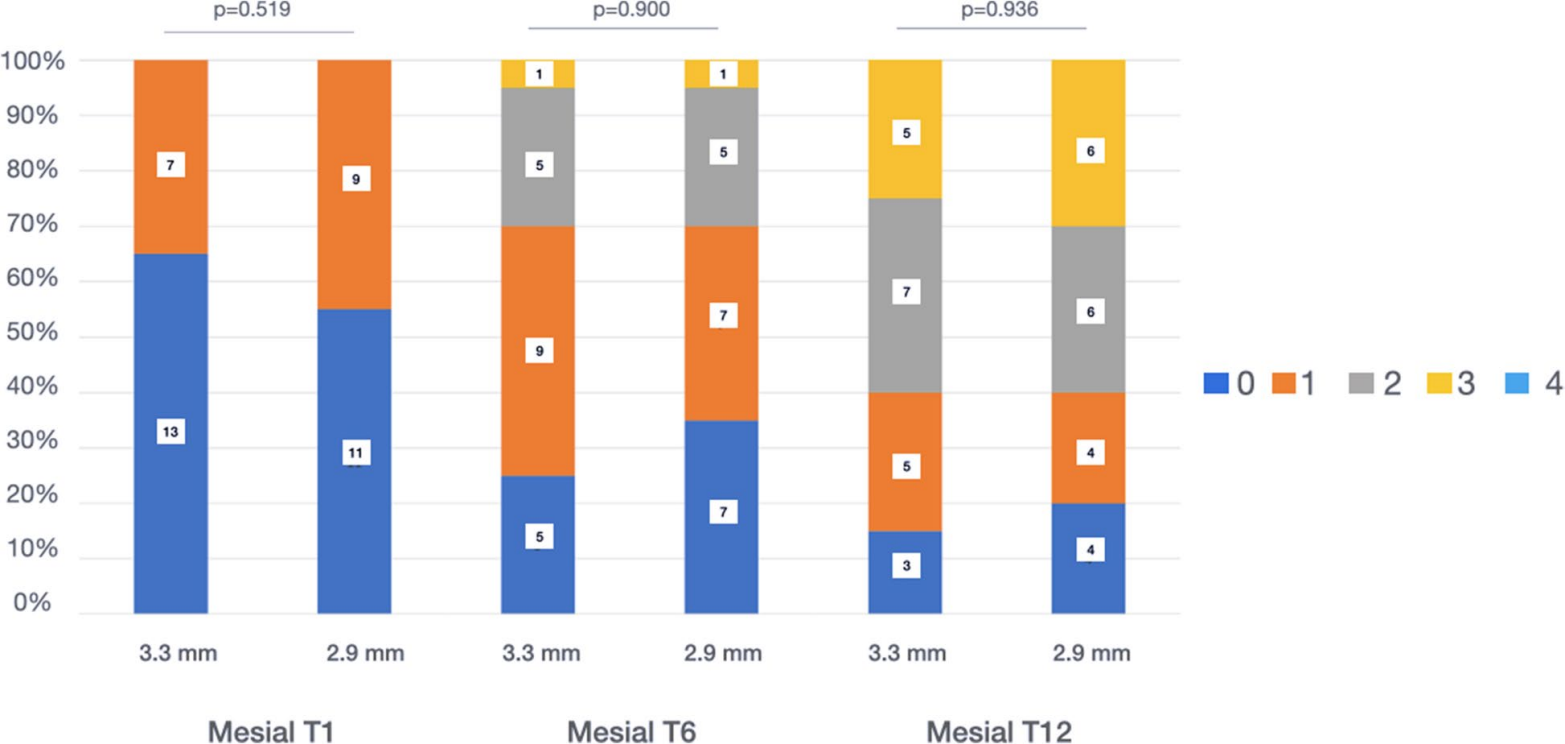
Mesial papilla JPI. T1: placement of the prosthesis; T6: 6 months; T12: 12 months

**Fig. 6:**
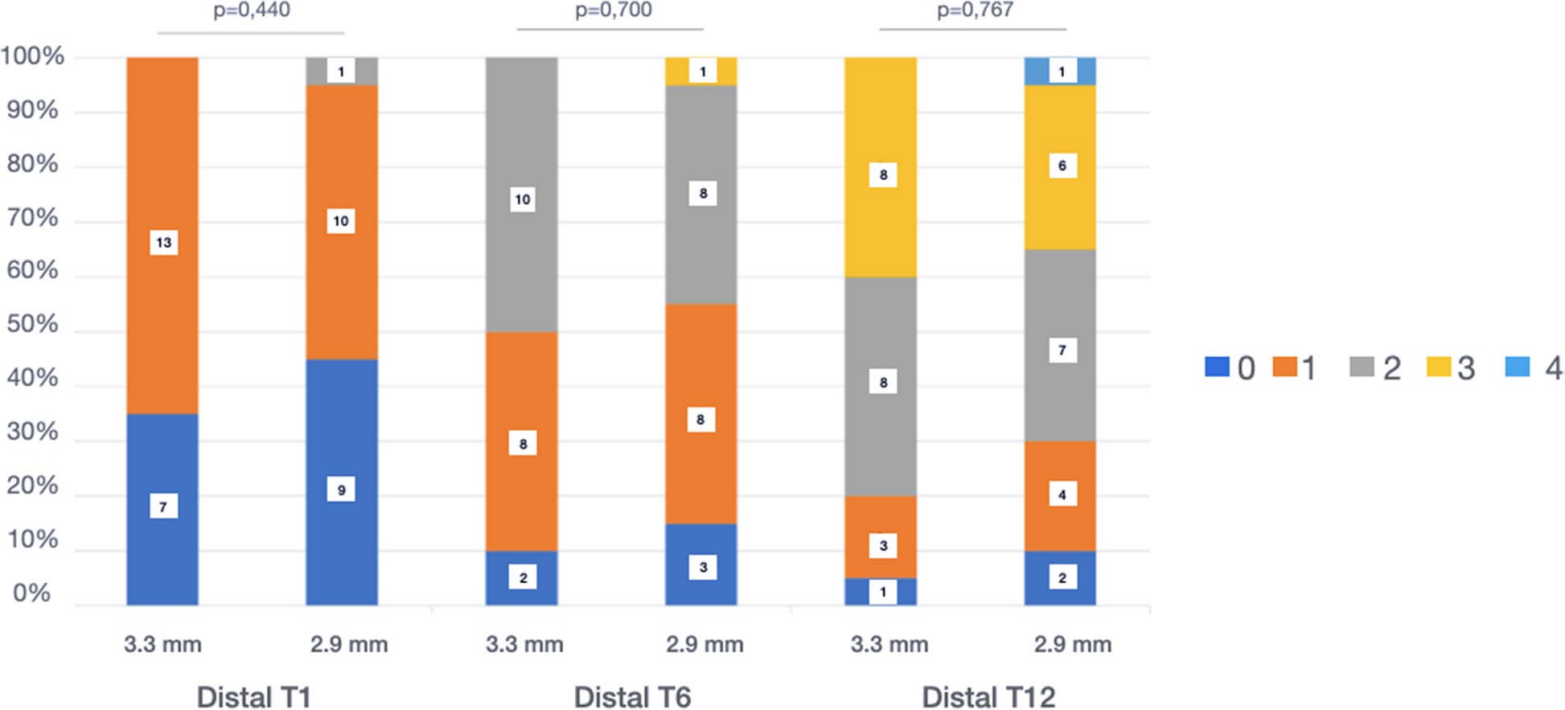
Distal papilla JPI. T1: placement of the prosthesis; T6: 6 months; T12: 12 months

At the beginning of the study, most patients had a JPI of 0 in the mesial (TG: 55% *vs.* CG: 65%) and a JPI of 1 in the distal location (TG: 50% *vs.* CG: 65%). During the follow-up, the formation of the soft tissue between the natural tooth and the NDI was observed in both groups, at both 6 and 12 months. At the end of the study (T12), complete papilla formation (JPI = 3) of the tooth-implant space was obtained mesially (TG: 30% *vs*. CG: 25%) and distally (TG: 40% *vs.* CG: 30%), with more papilla fill in the TG in both locations. In one patient in the TG, hyperplastic growth of the papilla (JPI = 4) was observed (Figs. 5 and 6).

Intergroup and intragroup analyses were performed in patients with a history of periodontitis (TG: 95% vs CG:65%). At baseline, no differences were observed in both groups at mesial locations or distal locations. Most of the patients showed a JPI = 0 at mesial locations (TG:52.63% vs CG:69.23%); at distal locations fewer patients showed this index (TG:47.37% vs CG:38.46%) and most patients in CG showed a JPI = 1 (TG:47.37% vs CG:61.54%), without any significant differences. At the end of the study, the majority of the patients showed a JPI ≥ 2 at mesial (TG:57.9% vs CG:38.46%) and at distal sites (TG:63.16% vs CG:63.16%), respectively. No significant differences were observed in papilla gain in either group (see Additional file 1).

In contrast, intragroup analysis performed in patients with a history of periodontitis or not, showed heterogeneous results in both locations. Whereas at the level of the distal papilla, there were no statistical differences in either the GC or the TG, at mesial locations there was a difference in the CG where all the patients without a history of periodontitis had a JPI ≥ 2 (*p* = 0.03)(see Additional file 2).

### Clinical parameters

Mean PD, MPI, and BOP were similar in both groups. At the 12-month visit, an increase in PD was observed in both groups, while MPI and BOP remained stable. However, no association was found between MPI and BOP. There was no suppuration on probing in any implant of the study (Table 1).

**Table 1:** Clinical parameters

	Test group Mean (SD)		Control group Mean (SD)		*p*	
	**T0**	**T12**	**T0**	**T12**	**T0**	**T12**
PPD (mm)	1.9 (0.5)	2.8 (0.6)	2.1 (0.5)	3.3(0.6)	0.142	**0.006**
BOP (%)	20.0 (1.9)	18.5 (2.2)	19.6 (2.5)	17.7 (2.7)	0.698	0.301
MPI (%)	17.8 (1.8)	18.1 (2.4)	17.8 (1.8)	17.4 (2.2)	0.142	**0.006**
Suppuration on probing (%)	0	0	0	0		

*SD* Standard deviation, *PPD* Peri-implant probing depth, *BOP* Bleeding on probing, *MPI* Modified plaque index

### Complications

No surgical complications were recorded. Regarding prosthetic complications, the loosening of the crown screw and a prosthetic screw were recorded in 1 patient in the TG and in two patients in the CG, respectively.

### Patient-reported outcome measures (PROMs)

Patient satisfaction (PROMs) was high in both groups for all the variables analyzed. The comparison between groups showed an overall trend for higher ratings in the TG, with significantly higher ratings for aesthetics, comfort, and self-esteem (*p* < 0.05) (Table 2). When analyzing the results according to gender, the degree of satisfaction in women was statistically lower in all the variables registered (Table 3).

**Table 2:** Patient-related outcome measures at 12 months follow-up

	Test group Mean (SD) [range]	Control group Mean (SD) [range]	*p*
Female	20	20	> 0.05
Male	20	20	> 0.05
Age	67.3 (8.5) [43–82]	59.3 (11.8) [39–84]	**0.013**
Chewing	8.9 (0.8) [8–10]	8.5 (0.9) [7–10]	0.174
Comfort	8.7 (1.1) [7–10]	7.7 (1.1) [5–10]	**0.024**
Esthetic	8.8 (1.1) [7–10]	8.1 (1.1) [6–10]	**0.049**
Phonation	8.9 (1.1) [6–10]	8.4 (1.1) [7–10]	0.183
Self-esteem	9.4 (0.7) [8–10]	8.7 (0.6) [8–10]	**0.004**

*SD* Standard deviation

**Table 3:** Patient-related outcome measures according to gender at 12 months follow-up

	Male Mean (SD)[range]	Female Mean (SD)[range]	*P*
Chewing	9.1 (0.8) [7–10]	8.3 (0.6) [7–9]	**0.002**
Comfort	8.7 (1.3) [5–10]	7.7 (0.9) [6–10]	**0.006**
Esthetic	9.1 (0.9) [6–10]	7.8 (0.8) [6–9]	**0.001**
Phonation	9.2 (0.8) [7–10]	8.1 (0,9) [6–10]	**0.001**
Self-esteem	9.5 (0.6) [8–10]	8.6 (0.5) [8–10]	**0.001**

*SD* Standard deviation

## Discussion

Aesthetics and patient satisfaction are considered key elements that must be evaluated for all treatments [33, 34]. In this study, there were no differences in the formation of soft tissue (papilla) of the tooth-implant space, implant SR, clinical peri-implant parameters, prosthetic complications, or patient satisfaction, except for aesthetics, comfort, and self-esteem, between the TG (2.9 mm) and the CG (3.3 mm) at 12 months. Considering the primary outcome (papilla formation between adjacent tooth and dental implant), H0 could not be rejected, as there were no differences between the two types of narrow implants.

Improvement of soft tissue in the tooth-implant space was observed throughout follow-up in both study groups, agreeing with previous studies [41–45] where the papilla height or papilla dimension was measured with the JPI [35]. Complete papillary fill was observed in at least one-third of the sample [43, 44] during the first year [42].

Age was observed as an independent risk factor for not achieving complete papilla fill [43]. Schropp et al. (2005) also observed that patients older than 52 years had a higher risk of presenting a JPI < 1 (interdental papilla height less than 50%) than younger patients (OR = 6.4, *p* = 0.03 mesially; OR = 9.3 *p* = 0.03 distally). In this study, patients in the TG (mean age: 67.3) obtained a JPI = 3 at 12 months of follow-up at mesial (30%) and distal (40%) location, respectively. Recently, narrow implants for the treatment of congenital agenesis of MLI in young patients were assessed and the complete fill of the papilla was observed in most of the patients [28]. This age difference and the history of periodontitis could explain the differences in papillary filling observed in the present study. In the apico-coronal dimension, it has been informed that a distance of 5 mm is necessary from the alveolar bone crest to the interdental contact point [14, 46]. When this distance is higher, papilla formation is possible at least 50% of the cases, but with no predictability [46]. Similar to the teeth, the distance of the bone peak to the interdental contact point (OR = 2.9) was associated to the risk of not achieving interdental papilla in the anterior region [47]. This could determine the presence or absence of a complete papilla formation between the natural tooth and the implant [14, 34, 44–48], as well as the horizontal position of the implant respecting the adjacent tooth [47, 48].

This would also explain the fact that the greatest formation of soft tissue (papilla) occurred in patients under 40 years of age, thus agreeing with the results of previous studies in younger populations [28, 42, 49].

With a JPI = 4, it is unclear whether improvement occurred (i.e. an increase from JPI = 3) or whether hyperplasia occurred due to errors in the design of the prosthesis, plaque accumulation [35], or the intake of drugs that may influence the excessive growth of keratinized mucosa [42]. As a matter of fact, Schropp et al. [43, 44] did not include level 4 in their results. In this study, the complete index was maintained and only 1 patient, who had a history of kidney transplantation and treatment with immunosuppressants (Tacrolimus), had a JPI = 4 (TG) at 12 months. It has been reported that this drug may have direct action on gingival fibroblasts [50], potentially explaining the hyperplasia in this patient. Although the evidence on the outcome of implant therapy in patients which have been received a solid organ transplants such as liver, kidney or combination of different organs (heart and liver transplant patients) is scarce [51–53] high rates of IS has been registered (98–100%). There are very few absolute medical contraindications to therapy with dental implants. Today, there is a high percentage of the population with systemic diseases such as diabetes or cardiovascular diseases, and implant therapy is not contraindicated in well-controlled systemically compromised patients [39]. It seems that the control of the degree of the systemic condition or disease might be more important than the nature of the disorder itself [39, 51]

The SR was 100%, consistent with the results of a recent study [28] and confirming the results in the literature [18, 54, 55], with SR of 94.7% for implants smaller than 3 mm and between 97.4 and 97.7 for implants > 3.3 mm. No differences were observed between smoking and nonsmoking patients, as did a recent study that analyzed the survival of NDIs in patients who were smokers [56]. However, these were short-term studies (6 and 12 months), and in this sample, the number of smokers (1 in the TG and 4 in the CG) was small.

When analyzing the ISQ values, a very high primary stability was observed (greater in the TG), which could be interpreted as a favorable situation for osseointegration. This difference between the two groups could be explained by the fact that all the implants that were placed in the lower incisors area had a diameter of 2.9 mm (13 in total), as the literature shows that the greatest primary stability is found at the level of the anterior mandibular incisors [57, 58]. The design of both implants used in this study (conical design and with a moderately rough surface) has been associated with favorable stability results [59–61], which could explain the high levels of stability in both groups.

Currently, PROMs provided for clinical results have increased in value and relevance. Most of the patients in the study showed high satisfaction with the aesthetic and functional results (TG: 8.94 *vs.* CG: 8.2), agreeing with results reported in the literature referring to narrow dental implants in the esthetic area [62]. By gender, women had the lowest satisfaction levels, as reported in previous studies [55]. One explanation for this finding might be that women report the most dissatisfaction with quality of life, specifically with oral aesthetics [63]. These data should be considered when planning a treatment. Older patients showed the highest satisfaction with treatment, probably because older patients have chewing and aesthetic problems and, therefore, their quality of life improves after implant treatment [64].

In this study, there were no surgical complications, contrary to what has been reported in the literature [65]. This difference could be due to performing a CBCT before surgery for all patients. The CBCT allows for assessing the three-dimensional disposition of the implant concerning the neighboring teeth and the actual bone volume of the patient for the placement of the implant.

NDIs have been associated with a higher risk of technical complications due to the smaller size of their components such as fractures [23] Due to this risk, Ti-Zr dental implants were developed to minimize this complication. The greater biomechanical strength of these NDI could explain the absence of fractures being similar to previous studies [56, 66]

In this study, the complications were limited to three cases of screw loosening that is, minor complications that were resolved quickly without affecting the survival [67]. These results are consistent with a systematic review [68] in which the loosening/fracture of the screw or abutment was 12.7% and 0.35%, respectively.

The mean age of the patients in this study was higher (TG: 67.3 vs. CG: 59.3 years) than in previous studies of NDIs in the anterior area, where the most common indication was the congenital agenesis of the lateral incisors [18, 28, 62, 69, 70]. Despite global demographic aging, dental implant treatments in patients over 55 years have increased in recent decades [2, 4]. Therefore, these treatments are common in older patients and more so in periodontal patients, as in this study.

This prospective controlled study shows some limitations. First, the design of the study itself, which could not be a randomized clinical trial due to the characteristics of the implants, as they must be tailored to the anatomical characteristics of each subject. This implies that the distribution of the location of the implants between the two groups was not homogeneous and was in strict compliance with the necessary anatomical requirements [47, 71]. Second, the population of the study was older than previously reported, and with clinical attachment loss, so complete papilla fill was not expected in the majority of these patients. Additionally, the study follow-up was short; therefore, the results should be interpreted with caution.

However, this study also has its strengths, as it is a study performed in a real clinical scenario in healthy periodontal patients with the absence of single maxillary lateral or inferior incisor, where the patient’s satisfaction was evaluated.

Despite the limitations and because of the results obtained, it could be concluded that the achievement of complete formation of soft tissue in the interdental space between the natural tooth and NDI (tooth-implant papilla) at 12 months might be possible. Also, there was no significant difference between 2.9 mm and 3.3 mm NDIs in the anterior area (MI/MxLI). NDIs could be an effective treatment alternative in narrow interdental spaces for implant-supported fixed rehabilitation in periodontal patients. In addition, the degree of satisfaction by patients with both implants was high, and the complications associated with the implants were minimal.

### Supplementary Information

**Supplementary Material 1.**

**Supplementary Material 2.**

## Data Availability

The data supporting the conclusions of this article is included within the article. The dataset is available from the corresponding author upon reasonable request.

## Abbreviations

UPV/EHU: University of the Basque Country
NDI: Narrow dental implants
TG: Test group
CG: Control group
JPI: Jemt papillary index
SR: Implant survival rate
PROM_s_: Patient-reported outcomes measures
ISQ: Implant stability quotient
SD: Standard deviation
MI/MxLI: Mandibular incisors and/or maxillary lateral incisors
CEIm-E: Euskadi drug research ethics committee
CBCT: Cone beam computed tomography
PPD: Peri-implant probing depth
BOP: Bleeding on probing
MPI: Modified plaque index
VAS: Visual analog scale
