# Supplementary material for: Clinical analysis of the tooth-implant papilla for two narrow-diameter titanium-zirconium implants in the anterior area: prospective controlled clinical study
Source: BMC Oral Health. 2024 Mar 5;24:310. doi: 10.1186/s12903-024-04075-2 (PMC10916199; doi:10.1186/s12903-024-04075-2)
Supplement: Supplementary file 1 — Supplementary Material 1. [file 12903_2024_4075_MOESM1_ESM.docx]

| Group | **mesial JPI_T0 n (%)** | | | | **mesial_JPI_T2 n (%)** | | | | | |
| --- | --- | --- | --- | --- | --- | --- | --- | --- | --- | --- |
|  | **0** | **1** | **2** | **p value** | **0** | **1** | **2** | **3** | **4** | **p value** |
| **TG**  **N=19** | 10 (52.63) | 9 (47.37) | 0 (0) | 0.348 | 4  (21.05) | 4 (21.05) | 6 (31.58) | 5  (26.32) | 0  (0) | 0.517 |
| **CG**  **N=13** | 9 (69.23) | 4 (30.77) | 0 (0) |  | 3  (23.08) | 5 (38.46) | 4 (30.77) | 1  (7.69) | 0  (0) |  |
| Group | **distal JPI_T0 n (%)** | | | | **distal_JPI_T2 n (%)** | | | | | |
|  | **0** | **1** | **2** | **p value** | **0** | **1** | **2** | **3** | **4** | **p value** |
| **TG**  **N=19** | 9 (47.37) | 9 (47.37) | 1 (5.26) | 0.572 | 2  (10.53) | 4 (21.05) | 7 (36.84) | 5  (26.32) | 1  (5.26) | 0.894 |
| **CG**  **N=13** | 5 (38.46) | 8 (61.54) | 0  (0) |  | 1  (7.69) | 2 (15.38) | 6 (46.15) | 4  (30.77) | 0 (0) |  |
| **Additional file 1.** Intergroup analysis of Jemt Papillary Index (JPI) in patients with history of periodontitis. TG: test group; CG: control group; n= number of patients; %: percentage; T0: baseline; T2: 12 months | | | | | | | | | | |
