# Supplementary material for: Clinical analysis of the tooth-implant papilla for two narrow-diameter titanium-zirconium implants in the anterior area: prospective controlled clinical study
Source: BMC Oral Health. 2024 Mar 5;24:310. doi: 10.1186/s12903-024-04075-2 (PMC10916199; doi:10.1186/s12903-024-04075-2)
Supplement: Supplementary file 2 — Supplementary Material 2. [file 12903_2024_4075_MOESM2_ESM.docx]

| **Group** | **History of Periodontitis** | **mesial JPI_T0 n (%)** | | | | **mesial JPI_T2 n (%)** | | | | | |
| --- | --- | --- | --- | --- | --- | --- | --- | --- | --- | --- | --- |
|  |  | **0** | **1** | **2** | **p value** | **0** | **1** | **2** | **3** | **4** | **p value** |
| **TG** | **No (n=1)** | 1  (100) | 0  (0) | 0 (0) | 0.985 | 0 (0) | 0 (0) | 0 (0) | 1  (100) | 0 (0) | 0.483 |
|  | **Yes (n=19)** | 10 (52.63) | 9 (47.37) | 0 (0) |  | 4 (21.05) | 4 (21.05) | 6 (31.58) | 5 (26.32) | 0 (0) |  |
| **CG** | **No (n=7)** | 4 (57.14) | 3 (42.86) | 0 (0) | 0.589 | 0 (0) | 0 (0) | 3 (42.86) | 4 (57.14) | 0 (0) | **0.03** |
|  | **Yes (n=13)** | 9 (69.23) | 4 (30.77) | 0 (0) |  | 3 (23.08) | 5 (38.46) | 4 (30.77) | 1 (7.69) | 0 (0) |  |
| **Group** | **History of Periodontitis** | **distal JPI_T0**  **n (%)** | | | | **distal JPI_T2 n (%)** | | | | | |
|  |  | **0** | **1** | **2** | **p value** | **0** | **1** | **2** | **3** | **4** | **p value** |
| **TG** | **No (n=1)** | 0  (0) | 1  (100) | 0 (0) | 0.666 | 0 (0) | 0 (0) | 0 (0) | 1 (100) | 0 (0) | 0.653 |
|  | **Yes (n=19)** | 9 (47.37) | 9 (47.37) | 1 (5.26) |  | 2 (10.53) | 4 (21.05) | 7 (36.84) | 5 (26.32) | 1 (5.26) |  |
| **CG** | **No (n=7)** | 2 (28.57) | 5 (71.43) | 0 (0) | 0.658 | 0 (0) | 1 (14.29) | 2 (28.57) | 4 (57.14) | 0 (0) | 0,64 |
|  | **Yes (n=13)** | 5 (38.46) | 8 (61.54) | 0 (0) |  | 1 (7.69) | 2 (15.38) | 6 (46.15) | 4 (30.77) | 0 (0) |  |
| **Additional File 2**. Intragroup analysis of Jemt Papillary Index (JPI) considering the history of periodontitis. TG: test group; CG: control group; n= number of patients; %: percentage; T0: baseline; T2: 12 months. | | | | | | | | | | | |
